# Disability prevalence-context matters: A descriptive community-based survey

**DOI:** 10.4102/ajod.v8i0.512

**Published:** 2019-08-14

**Authors:** Soraya Maart, Seyi Amosun, Jennifer Jelsma

**Affiliations:** 1 Department of Health and Rehabilitation Sciences, University of Cape Town, Cape Town, South Africa

**Keywords:** disability, prevalence, context, service planning, census, survey

## Abstract

**Background:**

There is increasing interest in the collection of globally comparable disability data. Context may influence not only the rates but also the nature of disability, thus locally collected data may be of greater use in service delivery planning than national surveys.

**Objectives:**

The objective of this article was to explore the extent to which two areas, both under-resourced but geographically and socially distinct, differed in terms of the prevalence and patterns of disability.

**Method:**

A cross-sectional descriptive survey design was utilised, using stratified cluster sampling in two under-resourced communities in the Western Cape, South Africa. Nyanga is an informal urban settlement in Cape Town and Oudtshoorn is a semi-rural town. The Washington Group Short Set of questions was used to identify persons with disabilities (PWD), and a self-developed questionnaire obtained socio-demographic information.

**Results:**

The overall prevalence of disability was 9.7% (confidence intervals [CIs] 9.7–9.8) and the proportion of PWD was significantly different between the two sites (Chi-Sq = 129.5, *p* < 0.001). In the urban area, the prevalence rate of any disability was 13.1% (CIs 12.0–14.3) with 0.3% (CIs 0.1–0.6) reporting inability to perform any function at all. In contrast, the semi-rural community had a lower overall prevalence rate of 6.8% (CIs 6.0% – 7.8%) but a higher rate of those unable to perform any function: 1% (CIs 0.07–1.4). Disability was associated with gender, age, unemployment and lower income status in both areas.

**Conclusion:**

Deprived areas tend to show higher disability prevalence rates than the National Census estimates. However, the discrepancy in prevalence and patterns of disability between the two under-resourced areas indicates the need for locally specific data when planning health interventions.

## Introduction

According to the World Report on Disability, data on disability should be collected to estimate the prevalence of disability, plan appropriate services and to monitor equalisation of opportunities for people with disabilities (World Health Organization & World Bank 2011), among other reasons. Acknowledging the poor access to rehabilitation services by people with disabilities in South Africa (Maart & Jelsma 2013), the Department of Health (DoH) in the Western Cape commissioned two surveys of disability to inform the planning of appropriate services in the province. Two under-resourced areas, Nyanga in the rapidly growing large city of Cape Town, and the other in a much smaller town that serves a farming community, Oudtshoorn, were chosen by the DoH as being representative of provincial diversity. This article reports on the findings of these surveys.

Determining disability prevalence is a complex exercise because of the various discourses associated with the understanding of disability. Before the advent of the International Classification of Functioning, Disability and Health (ICF) (World Health Organization 2001), disability data were collected and presented using a medical model which entailed simply counting the number of people with impairments, which may have resulted in the under-reporting of disability, specifically in low-income countries (Loeb, Eide & Mont 2008; Mont 2007a; World Health Organization). The introduction of the ICF and its conceptualisation of disability led to the realisation that prevalence would be greatly influenced by contextual differences across population groups, which may affect the degree of functional limitation experienced by people living with impairments. Contextual differences and the population profile could be used as criteria for prioritising access to health services (Department of Health 2015; National Planning Commission Department). Communities with limited resources should be prioritised in service planning to ensure their continued participation in society. The National Health Insurance White Paper has recognised the marginalisation of the majority of South Africans because of the Apartheid legacy, and therefore prioritises poorer communities in health service delivery provision (Department of Health 2017).

The ICF defines disability as:

an umbrella term for impairments, activity limitations and participation restrictions. It denotes the negative aspects of the interaction between a person’s health condition(s) and that individual’s contextual factors (environmental and personal factors). (World Health Organization 2001:3)

The ICF conceptual framework has been adopted by the United Nations (UN), and has become the standard for obtaining globally comparable disability data (World Health Organization 2001). The Washington Group (WG) Short Set of questions, developed to screen for disability, is based on the ICF framework and has been tested and used in South Africa and other countries (Loeb & Eide 2006; Madans et al. 2004; Palmer & Harley 2012; Schneider et al. 2009).

The Global Burden of Disease Report, based on country reports of mortality and loss of health because of disease and injury, estimated the global prevalence of disability to be 15%, with higher prevalence rates estimated for low-income countries (GBD 2010 Country Collaboration 2013). In contrast to these estimates, high-income countries have consistently reported greater disability prevalence rates compared to low-income countries (World Health Organization & World Bank 2011). There may be several reasons for this, some of which could be the increased longevity of the ageing population in high-income countries and better recording of morbidity and disability data (Palacois-Ceña 2013). The apparent under-reporting of disability in low-income countries has been attributed in part to the stigma associated with disability in some areas and reporting methodologies (Baskind & Birbeck 2005; Green 2003; RAVIM & Handicap International Mozambique 2010; Sorsdahl et al. 2012, Schneider 2009). This lower reported prevalence rate is evident in South Africa, where the National Census estimated the prevalence of disability to be 7.5% in 2011 of the total population (Statistics South Africa 2012). The highest prevalence of disability has been reported among those with lower income, particularly those who had no schooling (10%) compared to those who had post-secondary education (3%) (Statistics South Africa 2012). Black Africans, who generally reside in under-resourced communities, were still found to have the highest rate of disability (7.8%) in the 2011 census (Lehohla 2005; Statistics South Africa 2012). Disability prevalence in South Africa is still closely associated with race and development, with those communities who have experienced greatest disadvantage presenting with the highest disability prevalence (Statistics South Africa 2012). Rural provinces such as the Free State, North West and the Northern Cape have reported disability prevalence rates between 10% and 11% (Statistics South Africa 2014).

McDermot (2011) emphasised the need for unbiased disability prevalence measures to ensure accurate measures of disability to identify disparities and to measure changes over time (McDermott & Turk 2011). If data are to be useful for planning services, they must be relevant to the area in which the services are to be provided, as acknowledged in the National Development Plan (NDP) 2030, which recognises that ‘there is no one-size-fits-all approach’ (1) (Department of Health 2015). It is the contention of the authors that data from the National Census relating to disability may not be sufficiently nuanced to be used in service planning, as they may not capture all of the factors shaping service needs of different communities. The items related to disability are included as part of a large battery of questions and the respondents may not give these items due consideration. As context may influence not only the rates but also the nature of disability, locally collected data may be of greater use in service delivery planning than national surveys. The objective of this article was thus to explore the extent to which two areas, both under-resourced but geographically and socially distinct, differed in terms of the prevalence and patterns of disability.

## Research setting

As the influence of contextual factors was to be explored, two diverse sites were identified within the province: Nyanga, an urban township area in Cape Town, and Oudtshoorn, a town in a farming district. Both these areas are considered deprived, as measured in the National Multiple Deprivation Index Survey (Noble & Babita 2006). Although similar regarding low income and high unemployment rates, they differ in language, culture and infrastructure. Nyanga is one of the oldest townships in Cape Town, created specifically for black African migrant workers in the Apartheid era. Nyanga still has a majority black African isiXhosa-speaking population (98.9%). The area consists of different dwelling types which include brick houses, shacks (informal houses usually consisting of zinc or iron structures) and backyard housing (temporary structures erected on properties which have permanent structures) (Housing Development Agency 2012). Oudtshoorn, a town in a farming district, is home to a primarily Afrikaans-speaking population of mixed-race or Khoi-San ancestry (77%) who were also disadvantaged during the Apartheid era. Black Africans made up 12% of the population in Oudtshoorn (Housing Development Agency 2012). The inhabitants live primarily in brick houses. The results of the 2011 National Census (Table 1) indicate that among other differences, Oudtshoorn has a larger proportion of elderly people, a much lower population density, fewer female-headed households and more owner-occupied brick houses, compared to Nyanga.

**TABLE 1:** Comparison of the 2011 National Census results.

Variable	Oudtshoorn	Nyanga
Total population	95 933.0	57 996.0
Young (0–14)	28.7	27.3
Working age (15–64) %	64.2	69.3
Elderly (65+)	7.2	3.4
Dependency ratio	55.8	44.3
Sex ratio	91.8	93.9
Population density - persons/km^2^	27.0	18 775.0
No schooling aged 20+ %	4.5	3.0
Higher education aged 20+ %	6.7	5.8
Matric aged 20+ %	25.1	25.3
Number of households	21 910.0	15 993.0
Average household size	4.2	3.5
Female-headed households %	36.2	46.4
Formal dwellings %	88.5	67.3
Housing owned or paying off %	61.7	35.7
Weekly refuse removal %	78.0	92.0
Piped water inside dwelling %	74.5	53.5
Electricity for lighting %	85.3	95.3

## Methodology

### Sampling

Descriptive cross-sectional household surveys were conducted, and two-stage cluster sampling was used (Galway et al. 2012). A group of informants were identified through the randomised, stratified cluster sampling of households in the two areas. Stratification was based on the type of dwelling as identified in an aerial map. The households to be visited were selected by delineating and numbering equivalent in size areas on a Google map and then randomly selecting areas which appeared to consist chiefly of each type of dwelling, for example informal and formal, stand-alone houses and apartments. The number of dwelling type to be included was proportional to the census information (Housing Development Agency 2012). Excel Rand function was used to identify these clusters for inclusion. Data collection was begun at the corner dwelling on a randomly chosen block in each selected area, and continued along the street from there, visiting every alternate house. The informant was the head of the household or the eldest person available at the time of the visit. They were interviewed to identify people with disabilities; thus, proxy report was used to identify those with disability (Maart & Jelsma 2013).

The sample size was calculated using Epi-Info Stat Calc, Version 6. The 2001 census data were used (proposal submission for the research was submitted before the release of the 2011 census information), which reported the Oudtshoorn population to be 85 000 and that of Nyanga 58 723. As cluster sampling was used at the level of geographical area and household, a design effect of 1.5 was used (Skinner 1986). The estimated prevalence of disability was 5.2% (Statistics South Africa 2001). With these parameters, a sample of 2778 subjects in Oudtshoorn and 2753 in Nyanga was required (95% confidence intervals [CIs] and a 1.5% margin of error). Anticipating that the average household in this province consisted of 3.8 members in 2001 (Housing Development Agency 2012), the minimum number of households to be visited was approximately 900. To allow for non-responses, the final number of households to be visited was 1000 in each area.

Subjects of the surveys included all adults and children older than 5 years of age who were permanent residents in the identified household. Information was only gathered on older children as the WG Short Set is not appropriate for children under 5 years of age (Madans et al. 2004). Individuals who were visitors to the home or not permanent residents were excluded from the study.

### Instrumentation

A questionnaire was developed that included basic demographic details of all household members and the informants themselves (Maart & Jelsma 2013). This included income, education and employment questions. There were three sections concerned with demographic characteristics (e.g. age, education) social characteristics (e.g. household composition) and biomedical characteristics. The WG Short Set (six questions) was used to identify persons with disability (Madans et al. 2004). This instrument has been validated for use in South Africa, and has been used in the South African National Census since 2011 (Lehohla 2005; Schneider et al. 2009).

### Procedure

Home-based carers working in the respective communities were identified as research assistants to assist with data collection. The research assistants attended a training workshop on administering the questionnaire and a research supervisor from the community was appointed for quality assurance purposes (Maart & Jelsma 2013). Ethical approval for the study was obtained from the Human Research Ethics Committee at the University of Cape Town. Prior to conducting the interview, the study was explained to the informant and written and verbal consent was obtained. If for any reason, respondents in the identified household could not be interviewed, a further visit at an alternative time was attempted before exclusion of the household.

### Data analysis

Descriptive statistics were used throughout. The percentage prevalence of the different levels of disability was calculated, with the numerator being the number of people reported to have scored ‘some difficulty’, ‘a lot of difficulty’ and ‘unable to do’ based on the WG Short Set Questions. The denominator was the total number of household members reported on by the informants. The demographic details and disability prevalence and severity were compared between the two areas, using Chi-square, *t*-test or the Mann–Whitney U tests, depending on the nature and distribution of the data. Confidence level was set at 95%.

### Ethical considerations

The research assistants attended a training workshop on administering the questionnaire. A research supervisor from the community was appointed for quality assurance purposes. Ethical approval for the study was obtained from the Human Research Ethics Committee at the University of Cape Town (HREC number 098/2012) and all participants signed informed consent prior to participating in the interview.

## Results

A combined total of 2107 households were approached in the two areas: Nyanga and Oudtshoorn. The demographic details are presented in Table 2.

**TABLE 2:** Comparison of the demographic characteristics of informants from the two sites.

Variable	Total	Oudtshoorn	Nyanga	*p*-value Oudtshoorn versus Nyanga
Households approached	2107	950	1157	-
Households included	2058	945	1113	-
Refusals or missing	49	5	44	-
Chi-sq	-	-	-	24.66
*p*	-	-	-	< 0.001
Persons on whom data were collected (N)	7336	4028	3308	-
Number excluding children under 5 years	6637	3602	3035	-
Care dependency ratio (Children+Elderly) or adults†	-	0.47	0.25	-
Entire sample (excluding children > 5 years)				
Mean age	32.8	33.0	32.5	-
SD	18.0	19.6	15.9	-
Range	6–98 years	6–98 years	6–93 years	-
*N*	6637	3602	3035	-
*t*	-	-	-	1.11
*p*	-	-	-	0.267
Informants				
Mean age	45.8	50.1	42.9	-
SD	13.9	14.6	12.6	-
Range	18–93 years	13–98 years	19–93 years	-
*N*	2048	939	1109	-
Missing	10	6	4	-
*t*	-	-	-	−12.02‡
*p*	-	-	-	< 0.001‡
Informants: Men				
%	51	49.3	52.8	-
*N*	1030	443	587	-
Missing	24	23	1	-
Chi-Sq	-	-	-	4.53
*p*	-	-	-	0.033
Average household size (including young children)	3.6	4.3	3.0	-
Employed between 15 and 65 years				
%	38.8	28.0	49.9	-
*N*	5182	2623	2559	-
Chi-Sq	-	-	-	188.8
*p*	-	-	-	< 0.001
Household income per month				
Median category	R1000 – R2999	R1000 – R2999	R3000 – R4999	-
Range	< 1000 to > 13 000	(< 1000 to >13 000	< 1000 to > 13 000	-
*N*	1614	720	894	-
Missing	488	225	263	-
Adjusted Z	-	-	-	5.22
*p*	-	-	-	< 0.001

Dependency ratio = (Number of Children [0–15] + Number of Pensioners [> 65])/Number of working age 16–65. SD, standard deviation.

† , Definition of dependency ratio: The dependency ratio measures the percentage of dependent people (not of working age)/number of people of working age (economically active).

‡ , estimated with separate variances.

### Demographic characteristics of the two populations

Oudtshoorn informants were significantly older on average than those in Nyanga (*p* < 0.001). In Oudtshoorn, there were more female informants (50.7%) compared to Nyanga (48%). The mean age of the entire sample was 32.8 (SD 18.0) years and there was no significant difference in the mean age between the two areas. The average household size was greater and the care dependency ratio almost twice as high in Oudtshoorn (0.47) compared to Nyanga (0.25).

Some form of employment was reported by 38.8% of the total sample. A smaller proportion was employed in Oudtshoorn (28.0% compared to 49.9% in Nyanga). There were a large number of missing responses to the income question in both sites and the median income category of the total sample was R1000 – R2999 of those reporting. The Oudtshoorn respondents reported significantly less income than Nyanga respondents (*p* < 0.001).

### Disability prevalence

In all, 645 individuals screened positive for having difficulty in any one of the domains, a prevalence of 9.7% (CI 9.0% – 10.4%).

The demographic information of the surveyed sample who did not have disability is compared to that of the people with disability (PWD) in Table 3.

**TABLE 3:** Demographic information of those who screened positive for disability compared to those not reporting a disability (*N* = 6637).

Variable	Surveyed without disability *N* = 5992	Disability *N* = 645	Test statistics
Male gender			
*N*	2744	253	-
%	46.8	39.8	-
Chi-sq	-	-	11.4
*p*	-	-	0.001
Mean age	31.6	48.4	-
SD	16.8	19.2	-
*t*	-	-	−21.29†
*p*	-	-	< 0.001†
Employed	1926	105	-
%	44.0	19.5	-
*N*	4381	539	-
Age group > 15	-	-	-
Chi-sq	-	-	118.9
*p*	-	-	< 0.001
Median household income			
Category per month	R1000 – R2999	R1000 – R2999	-
Range	0– > R13 000	0– > R13 000	-
Adjusted Z	-	-	6.18
*p*	-	-	< 0.001

SD, standard deviation.

† , separate variances.

People with disability tended to be older (*p* < 0.05) and there were more women (*p* = 0.001), higher unemployment (*p* < 0.001) and a lower income (*p* < 0.001) in this group compared to people without disabilities. People with disability in Oudtshoorn were older than their peers in Nyanga (*p* = 0.033) and had a lower median income (*p* = 0.017) (Table 4).

**TABLE 4:** Demographics of people screening positive for disability across the two sites.

Variable	Oudtshoorn *N* = 244	Nyanga *N* = 401	Total *N* = 645	Test statistics
Male gender	95	158	-	-
%	40.3	39.6	47.0	-
Chi-sq	-	-	-	0.027
*p*	-	-	-	0.871
Mean age	50.5	47.1	-	-
SD	20.4	18.4	41.1	-
Range	6–91	6–93	0–93	-
*t*	-	-	-	2.14
*p*	-	-	-	0.032
Employed	29	76	105	-
%	16.2	20.4	19.0	-
Age group > 15	179	372	552	-
Chi-sq	-	-	-	1.368
*p*	-	-	-	0.242
Median income				
Category per month	R1000 – R2999	R3000 – R4999	R1000 – R2999	-
Range	0– > R13 000	0– > R13 000	0– > R13 000	-
Adjusted Z	-	-	-	2.38
*p*	-	-	-	0.017

SD, standard deviation.

**FIGURE 1:**
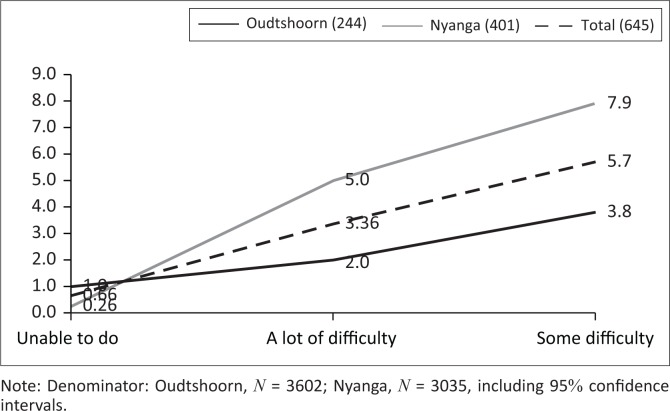
Percentage of total reported to have different levels of disability severity.

A significantly smaller proportion of respondents were identified as having a disability in Oudtshoorn (244, 6.8%, CI 5.9–7.6%) compared to Nyanga (401, 13.1%, CI 12.0% – 14.3%; Chi-sq = 77.8, *p* < 0.001) (Table 5, Figure 1). In addition, the CIs of the different severity levels did not overlap.

**TABLE 5:** Prevalence of disability in the two sites (Oudtshoorn *N* = 4028, Nyanga *N* = 3308).

Site	No difficulty	Some difficulty	Severe difficulty	Unable to do	Number with any disability	Number
**Oudtshoorn**	3358	137	71	36	244	3602
%	93.2	3.8	2.0	1.0	6.8	-
95% CI	92.3–94.0	3.2–4.5	1.6–2.5	0.7–1.4	6.0–7.8	-
**Nyanga**	2634	241	152	8	401	3035
%	86.8	7.9	5.0	0.3	13.1	-
95% CI	85.5–88.0	7.0–9.0	4.2–5.9	0.1–0.56	12.0–14.3	-
Totals	5992	378	223	44	645	6637
%	90.3	5.7	3.4	0.7	9.7	-
95% CI	89.5–91.0	5.2–6.3	2.9–3.8	0.60–0.7	9.7–9.8	-

CI, confidence interval.

There was a significant association between age group and presence of disability in the overall sample. The Oudtshoorn sample showed a relatively higher proportion of elderly and a lower proportion of youth among those with disabilities, compared to Nyanga (Table 6).

**TABLE 6:** Disability prevalence by age.

Variable	Youth (6–14 years)	Adult (15–59 years)	Elderly (60+ years)	Rowtotals
Oudtshoorn	14.0	169.0	61.0	244
Row (%)	5.7	69.3	25.0	-
Nyanga	17.0	324.0	60.0	401
Row (%)	4.2	80.8	15.0	-
**Totals**	**31**	**493**	**121**	**645**

Note: Chi-squared 11.49, *p* = 0.003.

## Discussion

The aim of this study was to compare disability prevalence and severity, together with demographic characteristics, in two under-resourced communities in the Western Cape Province, to determine whether more nuanced data on disability are required for service planning. It was the contention of the authors that inadequacies in service planning could result if National Census data only were relied upon.

### Sample

The study samples were representative of their respective districts and the Western Cape (Statistics South Africa 2012), in that they demonstrated a higher proportion of women compared to men in the population, and that the mean ages found in the study are comparable to the census of 2011 (Statistics South Africa 2012). The study sample was impoverished relative to the Western Cape at large, with an approximate average annual household income (R36 000, approximately $3600), which is much lower than the average household annual income recorded for the Western Cape (R143 461/$9647.59) (Statistics South Africa 2012). The annual income described in the study would translate to approximately $30 per month, which is less than $1 per day; this is way below the upper-bound poverty line of R992.

People identified with disability, who were more likely to be female and were older on average, have a higher unemployment rate and lower income than the general sample. The trend of increased disability among women is well documented globally and locally (Ethgen et al. 2004; Palmer & Harley 2012; Statistics South Africa 2012; Trani & Bakshi 2008). The higher incidence of non-communicable diseases, associated co-morbidities and increased life expectancy among women could account for the higher prevalence of women screening positive for disability (Peeters et al. 2013). The mean age of those screening positive for disability was older than the general sample which is not unexpected (Kostanjsek et al. 2013). The lower income associated with disability in this study is in contrast to that of Jelsma et al. (2007), who reported the household income to be higher in South African households with a disabled member and suggested that the disability grant available to those with disability could be the reason for this (Jelsma et al. 2008). It appears that in an urban setting, disability is associated with added financial advantage, as Nyanga reported a much higher median income for both the overall sample and among PWDs.

### Prevalence

The overall and site-specific prevalence rates in the study sample were higher than the average disability prevalence reported in the National Census (2011), despite the use of the same instrument to identify disability. As anticipated, the localised surveys revealed considerable differences between the two areas surveyed. Not only was there a variation in the overall prevalence of disability, the patterns differed with regard to levels of severity, ages and income of those with disabilities. While the overall rate of 9.7% is still lower than the WHO (2011) global estimate figure of 15%, the Oudtshoorn prevalence was 5% less than that of Nyanga. However, the prevalence of those who were ‘Unable to do’ although small was about three times higher in Oudtshoorn.

The disability prevalence in this study was found to be 4% – 5% for severe disability and 9% – 12% for moderate to mild functional limitations. These rates are similar to the World Health Survey (2013) (4% for severe and 16% for moderate to mild, respectively), and the Global Burden of Disease (2010) estimation (3% having extreme or severe disability and 15% having mild to moderate disability). The estimate for moderate to mild is somewhat lower (GBD 2010 Country Collaboration 2013; Ustun 2003). However, as hypothesised, the survey showed a much higher disability prevalence rate (9.7%) than reported in the South African census data (5.7%). The disability prevalence rates in both areas (Nyanga 13.1% and Oudtshoorn 6.8%) were higher than the Western Cape prevalence rate of 5.4% and comparable to less resourced provinces (Statistics South Africa 2014). These results may be partially explained by the continued deprivation and marginalisation experienced in disadvantaged communities in otherwise well-resourced provinces.

### Context

Apart from income and ethnic background, other aspects of the context and social environment are very different between the two areas. The burden of disability is borne by different groups in the two areas. In Nyanga, the mean age of those with disability was lower and 80% of those with disabilities were in the 15–59 year ‘Adult’ group. The increased prevalence of ‘some’ and ‘severe’ difficulties in this age group in Nyanga could be ascribed to the fact that Nyanga is described as one of the most violent neighbourhoods in the country, leading to high rates of trauma-related disability (South African Police Service). The high level of interpersonal violence is fuelled by rapid urbanisation and socioeconomic disparities which are much more evident in the urban context (Mont 2007b). These inequalities contribute greatly to the burden of disease and disability in South Africa (Norman et al. 2007). The extremely high population density of Nyanga (Table 1) (18 775 persons per square kilometre compared to only 27 persons in Oudtshoorn) may also be a factor. The impact of so many people living in a confined physical space is likely to manifest itself in pressure on facilities, lack of open spaces for recreation and invasion of personal space, all of which may contribute to the poor social climate evident in the high crime statistics (South African Police Service).

The difference in average age and household size between the sites could also be linked to urban migration. It is likely that those who become severely disabled often return to their families in rural areas, and this could explain why the proportion of people ‘unable to do’ is higher in Oudtshoorn (nearly three times as many) than in Nyanga. Another explanation could be lack of access to health services, which might be related to screening of health-related complications leading to more serious outcomes (Maart & Jelsma 2013). This, coupled with the higher care dependency ratio, indicates that caregivers in Oudtshoorn may well have difficulty in providing the required care to those with the most severe disability (Maart & Jelsma 2013). At the same time, those in Nyanga live in smaller households and they may not have someone who is able to aid and care. More research is thus required to adequately inform service delivery needs in the two areas.

### Implications of findings

Great emphasis has been placed on obtaining comparable data (Madans et al. 2004; Mont 2007a; World Health Organization 2001); however, the results of this study show that the simple reporting of only the prevalence of disability fails to capture the differences between communities. For services to be objectively prioritised, a more nuanced understanding of those presenting with disability is required. Although national or large-scale surveys such as the census can give general estimates of disability, these may not be useful for service planning. This study demonstrated that two areas, both of which are economically deprived, showed very different patterns of disability, both to one another and to provincial figures (Maart 2015). Locally specific information relating to persons with moderate to severe disability is of most relevance to policymakers and service providers, particularly in resource-constrained environments where there are competing priorities.

According to Helander (1992), it is reasonable to assume that persons with severe disabilities are dependent on others physically, economically, socially and psychologically, and would thus require a multidimensional approach to combat exclusion and facilitate their participation in society (Helander 1992). In both areas, our results indicate the need for continued financial support through disability grants and the creation of job opportunities, as PWDs appear to be less able to find employment and live in households with lower monthly income than those without disability.

### Study limitations

The major weakness of the study was that the estimates of disability were based on the proxy reporting of informants (Maart 2015). It is well known that proxy reports often result in under-identification of subjective problems (Maart 2015), such as mental and emotional problems, as these are not as readily apparent as physical or sensory disabilities. It is also acknowledged that the prevalence of depression and other mental disorders is likely to be under-reported as it is not included in the WG Short Set. A further limitation is that children younger than 5 years of age were excluded from this study as the WG Short Set has not been validated for this age range, and this gives a skewed view of disability and services required (Madans et al. 2004).

The number of refusals in Nyanga is a limitation that should be investigated as this group could have added to the disability prevalence.

A further limitation could also be that the census data of 2001 was used to estimate the sample size.

## Conclusions and recommendations

This study has demonstrated that small-scale surveys can provide more nuanced data on disability than National Census data. The higher disability prevalence rates reported in this study highlight the limitation of using National Census data in planning services for people with disabilities, in that the prevalence in the geographic areas could be underestimated. Inaccurate estimates mean that those who are not counted will remain invisible and will not reach their full potential. The different patterns of disability identified in the two study sites might well necessitate different approaches to the provision of rehabilitation services. It is recommended that the National Census be supplemented by smaller localised disability surveys for service planning.

The Western Cape Department of Health has been proactive in its community planning for people with disabilities and has used the results of this survey to inform policy on intermediate and community-based care. There is, however, a further need to provide rehabilitation services appropriate to the patterns of disability reported. For example, the Oudtshoorn community may benefit from home-based care services for those who are severely disabled. In contrast, in Nyanga, the disabilities are primarily in the wage-earning age group, and the focus should perhaps be on specialised rehabilitation for improvement in functional level and possible return to work. Services must also do more than provide rehabilitation – they must also address determinants of disability in under-resourced areas. Collaboration is needed between different sectors of government such as police, social development and health to ensure a safe and supportive environment for people with disabilities. Further research is required to establish the specific needs of people identified with disability living in different communities.

In conclusion, this study demonstrated the value of small-scale community-based surveys in providing more nuanced data for service planning. Disability prevalence alone does not highlight the needs of different age groups or across different levels of severity. This study is part of a PhD thesis focussing on disability in under-resourced areas in the Western Cape, South Africa (Maart 2015).
